# Effect of risk factors for acneiform rash induced by anti-epidermal growth factor receptor antibody drugs on survival: a retrospective observational study

**DOI:** 10.1186/s40780-022-00253-y

**Published:** 2022-09-01

**Authors:** Hiroaki Takahashi, Yukiko Yaegashi, Yoko Saito, Satoru Nihei, Tomohiko Tairabune, Haruki Ujiie, Junichi Asaka, Kenzo Kudo

**Affiliations:** 1 Division of Clinical Pharmaceutics and Pharmacy Practice, Department of Clinical Pharmacy, School of Pharmacy, Iwate Medical University, 1-1-1 Idaidori, Yahaba-cyo, Shiwa-gun, Iwate, 028-3694 Japan; 2 Department of Pharmacy, Iwate Medical University Hospital, 2-1-1 Idaidori, Yahaba-cyo, Shiwa-gun, Iwate, 028-3695 Japan; 3 Mayumi Pharmacy, 1-29-2 Shoto, Shibuya-ku, Tokyo, 150-0046 Japan; 4 Division of Integrated Information for Pharmaceutical Sciences, Department of Clinical Pharmacy, School of Pharmacy, Iwate Medical University, 1-1-1 Idaidori, Yahaba-cyo, Shiwa-gun, Iwate, 028-3694 Japan

**Keywords:** Colorectal cancer, Anti-EGFR antibody drug, Acneiform rash, High body weight, Survival

## Abstract

**Background:**

We previously reported that high body weight was a risk factor affecting the onset of anti-epidermal growth factor receptor (EGFR) antibody drug-induced acneiform rash. The current study investigated the relationship between risk factors for anti-EGFR antibody drug-induced acneiform rash and survival probability in colorectal cancer patients, as well as effects of drug withdrawal, dose reduction, or treatment discontinuation on treatment continuation.

**Methods:**

This retrospective study included 67 patients with unresectable advanced or recurrent colorectal cancer treated with anti-EGFR antibody drugs for the first time.

**Results:**

The survival time and acneiform rash grade of patients with high body weight (≥ 67.2 kg) were significantly longer and higher than those of patients with low body weight (< 67.2 kg). Moreover, the treatment continuation time of patients with drug withdrawal or dose reduction was significantly longer than that of patients without drug withdrawal or dose reduction or with/without treatment discontinuation. Meanwhile, the treatment continuation time of patients with treatment discontinuation was significantly shorter than that of patients with drug withdrawal or dose reduction or those without drug withdrawal, dose reduction, or treatment discontinuation.

**Conclusions:**

High body weight is a novel prognostic factor for patients receiving cancer drugs with anti-EGFR antibody drugs. Hence, the results of this study suggest that patients with high body weight should be carefully monitored for the development of acneiform rash when receiving anti-EGFR antibody drugs as cancer drug therapy.

## Background

With the advent of molecular-targeted agents, significant progress has recently been made in cancer drug treatment. In particular, cancer drug treatment using molecular-targeted agents has contributed to the prolonged survival time of patients with cancer [1, 2]. Molecular-targeted agents specifically target molecules involved in cancer cell proliferation, metastasis, and infiltration, thus having side effects that differ from conventional cancer drug treatments, characterized by nausea/vomiting, loss of appetite, stomatitis, and myelosuppression [3, 4].

Additionally, changes have been reported regarding the degree of distress among patients receiving cancer drug treatment. Although the advent of 5-HT_3_ receptor and neurokinin-1 receptor antagonists, as well as the establishment of the antiemetic drug guidelines, has reduced the occurrence of nausea and vomiting, the 2002 survey by Carelle et al. reported mental and social anxieties, including impact on family, partner, and society, as a common outcome in patients receiving cancer therapy [5]. Furthermore, cancer drug treatment has largely shifted from inpatient to outpatient, allowing several patients to receive treatment while working. Consequently, a 2013 survey by Nozawa et al. has shown that external physical symptoms, including hair loss, edema, and eczema, ranked high in patient distress [6]. However, the development of measures to counter these effects has proven challenging.

In particular, anti-epidermal growth factor receptor (EGFR) antibody drugs, which target EGFR, can cause the development of various skin disorders, such as acneiform rash, dry skin, and paronychia [7, 8], because EGFR is also expressed in normal skin. The skin disorders caused by these drugs not only cause psychological distress due to changes in appearance but also impact patients’ QOL [9]. Furthermore, considering that the degree of skin disorders caused by anti-EGFR drugs correlates with survival probability [1, 2], it is imperative that the skin disorders be properly controlled to avoid treatment discontinuation. Developing an effective means to predict the severity of skin disorders in patients prior to treatment with anti-EGFR antibody drugs will facilitate the timely administration of preventive or early measures.

Various risk factors have been described for acneiform rash. For example, male or young patients with colorectal cancer (CRC) treated with cetuximab, an anti-EGFR antibody drug, are at an increased risk of developing grade 2–3 acneiform rash [10]. Additionally, we previously reported high body weight as a risk factor for developing acneiform rash caused by anti-EGFR antibody drugs [11]. However, the effect of body weight on survival probability remains unclear. Therefore, this study retrospectively investigated the relationship between risk factors for developing acneiform rash during anti-EGFR antibody drug therapy and survival probability. We also investigated the effect of anti-EGFR antibody drug withdrawal, dose reduction, or treatment discontinuation on the probability of treatment continuation. The findings of this study may facilitate the implementation of preventive or early measures against skin disorders prior to treatment with anti-EGFR antibody drugs in patients who are expected to experience aggravation of skin disorders, thus controlling skin disorders and promoting treatment continuation. Hence, collectively, these results may lead to pain reduction, improvement in QOL, and prolongation of survival rates in cancer patients.

## Methods

### Target patients

We retrospectively examined the electronic medical records of patients with unresectable advanced or recurrent CRC who had received anti-EGFR antibody drugs (cetuximab or panitumumab) for the first time as cancer chemotherapy at Iwate Medical University Hospital from January 2014 to December 2018. Patients with skin diseases, including psoriasis or atopic dermatitis, were excluded from the study. This study included patients who were previously enrolled in a study that identified high body weight as a risk factor for developing acneiform rash during anti-EGFR antibody therapy [11]. Therefore, as in the previous study, the target patients were classified based on body weight, which was the risk factor for developing acneiform rash. The study was conducted in accordance with the Declaration of Helsinki and with the approval of the Ethics Committee of Iwate Medical University School of Medicine (approval number MH2021-107). This study was non-interventional and utilized a secondary data source; hence, no informed consent was required from the patients.

### Standard treatment

The standard treatments for patients with unresectable advanced or recurrent CRC that were examined in this study included cetuximab (Cmab)/panitumumab (Pmab) + FOLFOX (*l*-LV + bolus 5-FU + infusional 5-FU + L-OHP) therapy and Cmab/Pmab + FOLFIRI (*l*-LV + bolus 5-FU + infusional 5-FU + CPT-11) therapy.

The treatment schedule for each regimen was as follows: Cmab + FOLFOX therapy (Cmab, 400 mg/m^2^ initial dose, followed by 250 mg/m^2^ Day 1, 8; *l*-LV, 200 mg/m^2^ Day 1; bolus 5-FU, 400 mg/m^2^ Day 1; infusional 5-FU, 2400 mg/m^2^ Day 1–2; L-OHP, 85 mg/m^2^ Day 1; 2 weeks per cycle), Pmab + FOLFOX therapy (Pmab, 6 mg/kg Day 1; *l*-LV, 200 mg/m^2^ Day 1; bolus 5-FU, 400 mg/m^2^ Day 1; infusional 5-FU, 2400 mg/m^2^ Day 1–2; L-OHP, 85 mg/m^2^ Day 1; 2 weeks per cycle), Cmab + FOLFIRI therapy (Cmab, 400 mg/m^2^ initial dose, followed by 250 mg/m^2^ Day 1, 8; *l*-LV, 200 mg/m^2^ Day 1; bolus 5-FU, 400 mg/m^2^ Day 1; infusional 5-FU, 2400 mg/m^2^ Day 1–2; CPT-11, 150 mg/m^2^ Day 1; 2 weeks per cycle), and Pmab + FOLFIRI therapy (Pmab, 6 mg/kg Day 1; *l*-LV, 200 mg/m^2^ Day 1; bolus 5-FU, 400 mg/m^2^ Day 1; infusional 5-FU, 2400 mg/m^2^ Day 1–2; CPT-11, 150 mg/m^2^ Day 1; 2 weeks per cycle).

### Survey items and methods

The survey items included basic patient information (sex, age, cancer stage, height, body weight, medical history, chemotherapy history, and concomitant medications), blood biochemical tests (alanine aminotransferase [ALT], aspartate aminotransferase [AST], total bilirubin [T-Bil], and serum creatinine [Scr]), skin disorder onset status (degree of acneiform rash), usage status of external preparations (name of drug, such as moisturizer, and dosage), usage status of anticancer drugs (name of drugs and dosage), and survival time. These data were extracted from electronic medical or pharmaceutical care instruction records.

### Evaluation of skin disorders

Information on the presence and degree of acneiform rash—a characteristic observed during treatment with anti-EGFR antibody drugs—was collected from electronic medical records. Data on skin disorder grade, collected from treatment initiation to completion, was evaluated based on the Common Terminology Criteria for Adverse Events (CTCAE) used in cancer chemotherapy. Additionally, considering that acneiform rash generally develops within 1 month of EGFR administration [12], the onset status of acneiform rash was evaluated 1 month after drug administration. Information on drug withdrawal, dose reduction, or treatment discontinuation was collected from treatment initiation to completion.

### Evaluation of treatment continuation and survival probability

Treatment continuation probability was defined as the period from the start of treatment with anti-EGFR antibody drugs to any change in regimen. Survival probability was defined as the period from the day of cancer diagnosis to the day of death or discontinuation due to any cause.

### Statistical analysis

The chi-squared test, Student’s *t*-test, and Fisher’s exact test were used to analyze patient characteristics for each group classified based on body weight. Propensity score matching was also used to adjust for patient background bias when classifying patients into two groups based on body weight. The Kaplan–Meier method (log-rank test) was used to compare the survival probability for each group, classified by body weight. In addition, the Mann–Whitney *U* test was used to compare the acneiform rash grade for each group, classified by body weight. Moreover, a receiver operating characteristic (ROC) curve was constructed to set the optimum cutoff value, and the target patients were classified into two groups.

The chi-squared test and one-way analysis of variance (ANOVA) were used to analyze patient characteristics for each group, classified by the presence or absence of drug withdrawal, dose reduction, or treatment discontinuation of anti-EGFR antibody drugs. Tukey’s honest significant difference test was used to analyze multiple comparisons after a one-way ANOVA. The Kaplan–Meier method (log-rank test) was used to compare the probability of treatment continuation for each group, classified by the presence or absence of drug withdrawal, dose reduction, or treatment discontinuation of anti-EGFR antibody drugs. SPSS Statistics ver. 28 (IBM, Armonk, NY) was used for all statistical analyses, and results with *p* values of < 0.05 were considered statistically significant.

## Results

### Target patients

During the study period, 67 patients with unresectable advanced or recurrent CRC received anti-EGFR antibody drugs as cancer chemotherapy for the first time; none of them reported skin diseases, such as psoriasis or atopic dermatitis. The characteristics of the 67 patients are presented in Table 1. Moreover, 18 and 49 patients received cetuximab and panitumumab, respectively, as anti-EGFR antibody drugs. The treatment regimens used were Cmab + FOLFIRI therapy in 10 patients, Cmab + FOLFOX therapy in 8 patients, Pmab + FOLFIRI therapy in 26 patients, and Pmab + FOLFOX therapy in 23 patients. All patients used heparinoid moisturizers, and 37 patients used minocycline as supportive care for acneiform rashes caused by anti-EGFR antibody drugs.

**Table 1:** Patient characteristics (*n* = 67)

Characteristic	Value
Sex (male/female)	37/30
Age (years)	62.5 ± 1.39
Anti-EGFR antibody drug	
Cetuximab	18
Panitumumab	49
Regimen	
Cmab + FOLFIRI therapy	10
Cmab + FOLFOX therapy	8
Pmab + FOLFIRI therapy	26
Pmab + FOLFOX therapy	23
Moisturizer	
Heparinoid	67
Supportive care drug	
Minocycline	37
Physical information	
Height (cm)	163.4 ± 0.99
Body weight (kg)	60.4 ± 1.46
Blood biochemical data	
AST (IU/L)	29.2 ± 2.89
ALT (IU/L)	23.3 ± 2.57
T-Bil (mg/dL)	0.52 ± 0.04
Scr (mg/dL)	0.77 ± 0.03

Values are presented as mean ± standard error

*AST* aspartate aminotransferase, *ALT* alanine aminotransferase, *T-Bil* total bilirubin, *Scr* serum creatinine

### Effects of acneiform rash risk factors on survival

The target patients were classified into two groups based on body weight, extracted as a risk factor from our previous report [11]. Patient characteristics for each group, classified by body weight, are shown in Table 2. According to body weight, the target patients were classified into low body weight (< 67.2 kg) and high body weight (≥ 67.2 kg) groups to compare each factor. The results showed a higher proportion of males than females and significantly higher heights in patients with high body weight compared to those with low body weight. However, no differences were detected between the groups in cancer stage or in using minocycline, which provides supportive care for acneiform rash caused by anti-EGFR antibody drugs. After propensity score matching, there were 14 patients with high body weight and 14 patients with low body weight, with no differences in patient background characteristics, such as sex or height, between the two groups.

**Table 2:** Patient characteristics for each group classified based on body weight

	**Before propensity score matching**				**After propensity score matching**		
**Characteristic**	**Low** **body weight** ** < 67.2 kg** **(*n* = 53)**	**High** **body weight** ** ≥ 67.2 kg** **(*n* = 14)**	***p*-value**	**Low** **body weight** ** < 67.2 kg** **(*n* = 14)**		**High** **body weight** ** ≥ 67.2 kg** **(*n* = 14)**	***p*-value**
Sex (male/female)	25/28	12/2	0.010^a^*	9/5		12/2	0.385^*c*^
Age (years)	62.9 ± 1.50	61.3 ± 3.57	0.652^*b*^	61.7 ± 4.02		61.3 ± 3.57	0.937^*b*^
Anti-EGFR antibody drug (Cetuximab/Panitumumab)	15/38	3/11	0.743^*c*^	4/10		3/11	1.000^*c*^
Cancer stage (III/IV)	9/44	1/13	0.675^*c*^	2/12		1/13	1.000^*c*^
Height (cm)	162.0 ± 0.98	168.7 ± 2.58	0.005^b^*	165.5 ± 1.21		168.7 ± 2.58	0.267^*b*^
AST (IU/L)	28.2 ± 2.77	33.4 ± 9.20	0.468^*b*^	31.5 ± 7.07		33.4 ± 9.20	0.874^*b*^
ALT (IU/L)	22.9 ± 3.17	24.8 ± 2.76	0.762^*b*^	22.6 ± 2.79		24.8 ± 2.76	0.590^*b*^
T-Bil (mg/dL)	0.53 ± 0.05	0.50 ± 0.04	0.782^*b*^	0.44 ± 0.04		0.50 ± 0.04	0.318^*b*^
Scr (mg/dL)	0.74 ± 0.03	0.86 ± 0.06	0.098^*b*^	0.85 ± 0.05		0.86 ± 0.06	0.822^*b*^
Dosage of moisturizer for 1 month after initiation (g)	176.6 ± 13.6	167.9 ± 28.2	0.772^*b*^	144.6 ± 13.9		167.9 ± 28.2	0.469^*b*^
Minocycline treatment (Absence/Presence)	23/30	7/7	0.659^*a*^	5/9		7/7	0.445^*a*^

Values are presented as mean ± standard error

*AST* aspartate aminotransferase, *ALT* alanine aminotransferase, *T-Bil* total bilirubin, *Scr* serum creatinine

^a^chi-squared test

^b^Student’s *t*-test

^c^Fisher’s exact test

^*^p < 0.05

**Fig. 1:**
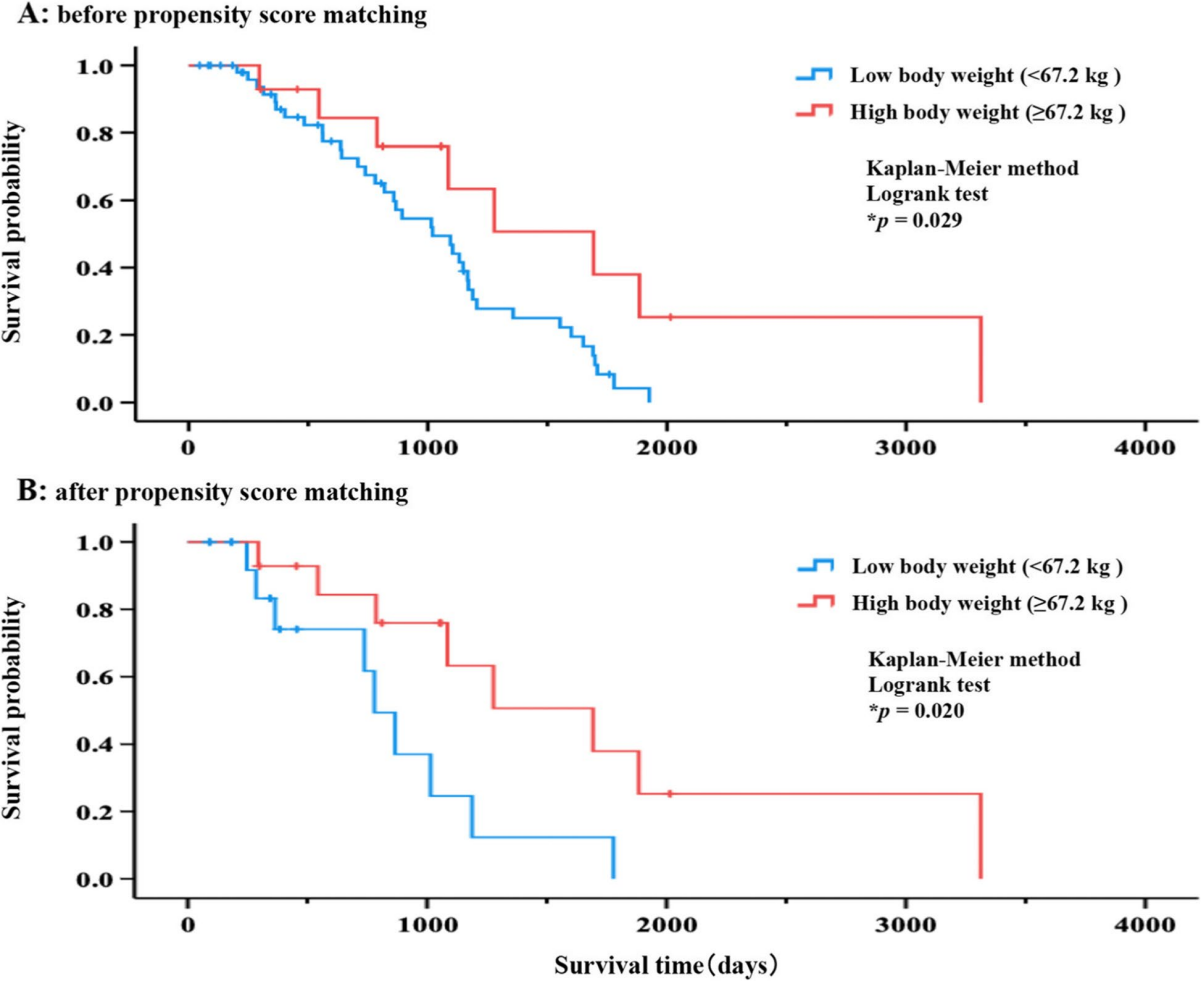
Comparison of survival probability. **A** and **B** show survival probabilities before and after propensity score matching, respectively. The Kaplan–Meier method (log-rank test) was used to compare the survival probability of the groups, which were classified by body weight. Patients with low body weight (< 67.2 kg) are represented with a blue line, whereas patients with high body weight (≥ 67.2 kg) are indicated by a red line. The median survival times for patients with low and high body weight were 1020 days (95% confidence interval [CI], 738–1302) and 1693 days (95% CI, 910–2476), respectively (**A**). Furthermore, the results after propensity score matching were similar to those before propensity score matching, with significantly longer survival time in patients with high body weight (**B**). *p < 0.05

Subsequently, the survival probabilities for each group classified by body weight were compared. The median survival time for patients with high body weight was significantly longer than that for patients with low body weight (1693 days [95% confidence interval, CI, 910–2476] vs 1020 days [95% CI, 738–1302], p = 0.029) (Fig. 1A). Similarly, after propensity score matching, patients with high body weight had significantly longer survival time than those with low body weight (*p* = 0.020) (Fig. 1B). The median survival times for patients with low body weight and high body weight were 781 days (95% CI, 612–950) and 1693 days (95% CI, 910–2476), respectively (Fig. 1B).

### Relationship between body weight and acneiform rash grade

The results of the comparison of acneiform rash grades for each group, classified based on body weight, are shown in Table 3. Target patients were classified into low or high body weight groups, and each factor was compared between groups. The acneiform rash grade was significantly higher in patients with high body weight than in those with low body weight.

**Table 3:** Comparison of acneiform rash grade for each group classified based on body weight (*n* = 67)

	**Low body weight** ** < 67.2 kg** **(*n* = 53)**	**High body weight** ** ≥ 67.2 kg** **(*n* = 14)**	***p*-value**
Acneiform rash grade (1 month)	0.94 ± 0.09	1.57 ± 0.17	0.002^a*^

Values are presented as mean ± standard error

^a^Mann–Whitney *U* test

^*^p < 0.05

### Impact of anti-EGFR antibody drug withdrawal, dose reduction, or treatment discontinuation on the probability of treatment continuation

Patient characteristics for each group, classified based on the presence or absence of anti-EGFR antibody drug withdrawal, dose reduction, or treatment discontinuation, are shown in Table 4. Target patients were classified as those without drug withdrawal, dose reduction, or treatment discontinuation; with drug withdrawal or dose reduction; or with treatment discontinuation for comparison of each factor. The results showed a higher proportion of males than females, as well as significantly higher height values, in patients with drug withdrawal or dose reduction compared to those without drug withdrawal, dose reduction, or treatment discontinuation (Tukey’s HSD test, *p* = 0.018). In addition, the body weights of patients with drug withdrawal or dose reduction and those with treatment discontinuation were significantly higher than those without drug withdrawal, dose reduction, or treatment discontinuation (Tukey’s HSD test, *p* = 0.007 and *p* = 0.006, respectively). Therefore, the three groups differed in sex, height, and body weight. However, no differences were observed in the usage status of minocycline.

**Table 4:** Patient characteristics for each group classified based on the presence or absence of anti-EGFR antibody drug withdrawal, dose reduction, or treatment discontinuation (*n* = 67)

Characteristic	Without drug withdrawal, dose reduction, or treatment discontinuation (*n* = 56)	With drug withdrawal or dose reduction (*n* = 7)	With treatment discontinuation (*n* = 4)	*p*-value
Sex (male/female)	27/29	7/0	3/1	0.024^a*^
Age (years)	63.2 ± 1.48	61.7 ± 3.40	55.0 ± 9.50	0.382^*b*^
Anti-EGFR antibody drug (Cetuximab/Panitumumab)	15/41	2/5	1/3	0.991^*a*^
Height (cm)	162.5 ± 0.98	171.2 ± 2.44	162.8 ± 7.66	0.025^b*^
Body weight (kg)	57.9 ± 1.22	71.5 ± 6.29	75.7 ± 9.45	< 0.001^b*^
AST (IU/L)	29.9 ± 3.37	27.6 ± 5.96	22.5 ± 4.35	0.820^*b*^
ALT (IU/L)	22.4 ± 2.96	29.7 ± 5.39	24.3 ± 7.38	0.687^*b*^
T-Bil (mg/dL)	0.50 ± 0.05	0.69 ± 0.15	0.50 ± 0.07	0.405^*b*^
Scr (mg/dL)	0.76 ± 0.03	0.90 ± 0.06	0.68 ± 0.10	0.254^*b*^
Dosage of moisturizer for 1 month after initiation (g)	179.3 ± 13.4	189.3 ± 44.6	106.3 ± 21.3	0.348^*b*^
Minocycline treatment (Absence/Presence)	28/28	1/6	1/3	0.144^*a*^

AST aspartate aminotransferase, *ALT* alanine aminotransferase, *T-Bil* total bilirubin, *Scr* serum creatinine

Values are presented as mean ± standard error

^a^chi-squared test

^b^one-way ANOVA

^*^p < 0.05

**Fig. 2:**
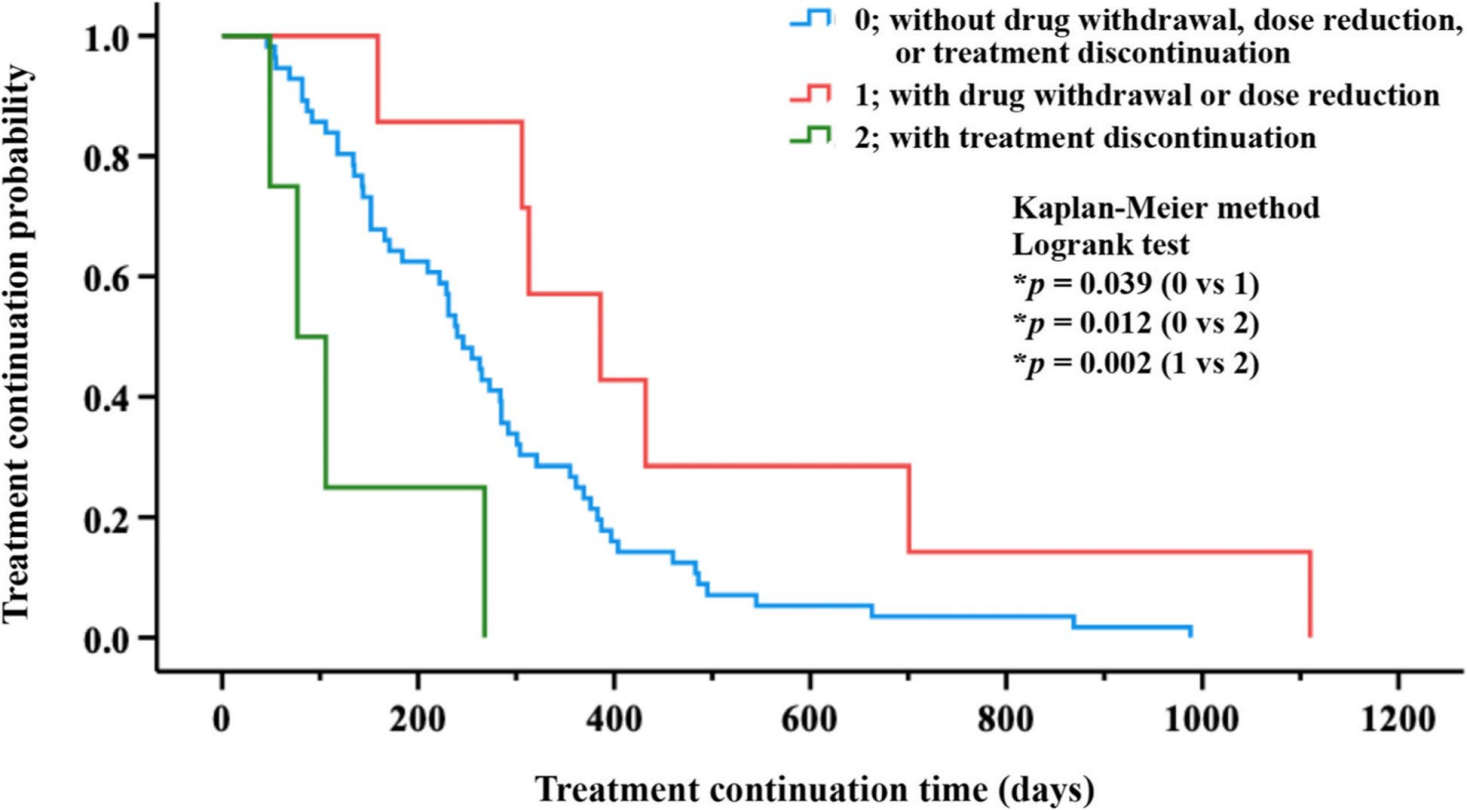
Comparison of the probability of treatment continuation. The Kaplan–Meier method (log-rank test) was used to compare the probability of treatment continuation of the groups, which were classified by the presence or absence of anti-EGFR antibody drug withdrawal, dose reduction, or treatment discontinuation. Patients with treatment discontinuation are represented with a green line; patients without drug withdrawal, dose reduction, or treatment discontinuation are represented with a blue line; and patients with drug withdrawal or dose reduction are represented with a red line. The median survival times for patients with treatment discontinuation; without drug withdrawal, dose reduction, or treatment discontinuation; and with drug withdrawal or dose reduction were 77 days (95% confidence interval [CI], 21–133), 240 days (95% CI, 204–276), and 386 days (95% CI, 199–573), respectively. *p < 0.05

Then, the probability of treatment continuation for each group classified based on the presence or absence of anti-EGFR antibody drug withdrawal, dose reduction, or treatment discontinuation was compared. The median survival times for patients with treatment discontinuation; without drug withdrawal, dose reduction, or treatment discontinuation; and with drug withdrawal or dose reduction were 77 days (95% CI, 21–133), 240 days (95% CI, 204–276), and 386 days (95% CI, 199–573), respectively. Moreover, the treatment continuation time of patients with drug withdrawal or dose reduction was significantly longer than that of patients without drug withdrawal, dose reduction, or treatment discontinuation or with treatment discontinuation (Fig. 2). Meanwhile, the treatment continuation time of patients with treatment discontinuation was significantly shorter than that of patients with drug withdrawal or dose reduction or without drug withdrawal, dose reduction, or treatment discontinuation (Fig. 2).

## Discussion

This study retrospectively investigated the relationship between risk factors for acneiform rash caused by anti-EGFR antibody drugs and survival probability and the effect of drug withdrawal, dose reduction, or treatment discontinuation on treatment continuation probability. First, we investigated the effect of body weight—a risk factor for acneiform rashes—on survival. The survival time of patients with high body weight (≥ 67.2 kg) was significantly longer than that of patients with low body weight (< 67.2 kg; Fig. 1A and 1B). Moreover, we investigated the relationship between body weight and acneiform rash grade and found a significantly higher acneiform rash grade in patients with high body weight than in patients with low body weight (Table 3). Previous studies have reported that a higher acneiform rash grade correlates with a higher survival probability [1, 2]. Therefore, in this study, it was suggested that acneiform rash caused by anti-EGFR antibody drugs was more likely to occur in patients with high body weight; hence, survival probability was higher in this patient group.

The increased occurrence of acneiform rash in patients with high body weight is likely due to patients with a high body mass index (BMI) reportedly having high transepidermal water loss (TEWL) [13], which is an index of skin barrier function. This means that a high TEWL value indicates reduced skin barrier function. In the current study, as body weight increased, BMI also generally increased, resulting in a significantly higher BMI value in patients with high body weight (27.7 ± 0.92) than in those with low body weight (21.2 ± 0.33, Student’s *t*-test, *p* < 0.001). Thus, skin barrier function may have been reduced in these patients with high body weight, potentially increasing their susceptibility to skin disorders caused by anti-EGFR antibody drugs. Considering the retrospective nature of this study, the TEWL values were not measured; however, they will be further investigated in future studies.

Furthermore, we investigated the effect of anti-EGFR antibody drug withdrawal, dose reduction, or treatment discontinuation on the probability of treatment continuation. Results showed that the treatment continuation time of patients with drug withdrawal or dose reduction was significantly longer than that of patients without drug withdrawal, dose reduction, or treatment discontinuation or with treatment discontinuation (Fig. 2). However, the treatment continuation time of patients with treatment discontinuation was significantly shorter than that of patients with drug withdrawal or dose reduction or without drug withdrawal, dose reduction, or treatment discontinuation (Fig. 2). In addition, the body weights of patients with drug withdrawal or dose reduction or with treatment discontinuation were significantly higher than those of patients without drug withdrawal, dose reduction, or treatment discontinuation (Table 4). These results suggest that both patients with drug withdrawal or dose reduction and those with treatment discontinuation may develop severe acneiform rash due to increased body weight. Moreover, failure to control skin symptoms may result in treatment discontinuation and a shorter treatment continuation time. In contrast, successful control of skin symptoms by drug withdrawal or dose reduction may result in avoidance of treatment discontinuation and a longer treatment continuation time.

In addition to anti-EGFR antibody drugs, bevacizumab [14, 15], an anti-vascular endothelial cell growth factor antibody drug, and regorafenib [16], a multikinase inhibitor, are standard treatments for patients with unresectable advanced or recurrent CRC. A characteristic side effect of regorafenib is hand-foot skin reaction. Various studies have reported on the relationship between the grade of hand–foot skin reaction caused by regorafenib and survival time [17, 18] and the prognostic factors that affect survival time [18]. In particular, a Phase IIb study on patients with metastatic CRC treated with regorafenib reported that patients with ≥ grade 2 hand–foot skin reaction have longer survival times than patients with grade 0–1 hand–foot skin reaction [17]. Moreover, in a large prospective regorafenib study in metastatic CRC, prolonged survival was reported to be associated with early (28 days) hand–foot skin reaction and good performance status (0–1) [18]. These findings may correlate with an increased incidence of hand–foot skin reactions in individuals with active lifestyles who exhibit good performance status, affecting survival time [18]. Moreover, in this previous study, a large body surface area (≥ 1.6 m^2^) was identified as a prognostic factor that affects survival time; however, this point was not further interpreted by the authors. Meanwhile, in the current study, it was impossible to investigate the effects of specific cancer drugs (such as regorafenib) following the administration of anti-EGFR antibody drugs. Therefore, a more detailed study is required in the future. Additionally, considering the small patient population from a single medical facility, the results described here must be further validated in a larger, multi-center, prospective study.

## Conclusions

The results of this study suggest that patients with high body weight should be carefully monitored for the development of acneiform rash when receiving anti-EGFR antibody drugs as cancer drug therapy.

## Data Availability

All data generated or analyzed in this study are included in the manuscript.

## Abbreviations

EGFR: Anti-epidermal growth factor receptor
CRC: Colorectal cancer
Cmab: Cetuximab
Pmab: Panitumumab
CTCAE: Common Terminology Criteria for Adverse Events
